# Longitudinal associations between reading for pleasure and child maladjustment: Results from a propensity score matching analysis

**DOI:** 10.1016/j.socscimed.2020.112971

**Published:** 2020-05

**Authors:** Hei Wan Mak, Daisy Fancourt

**Affiliations:** Department of Behavioural Science and Health, University College London, UK

**Keywords:** Reading for pleasure, Strengths and difficulties (SDQ), Child development, Longitudinal study, Propensity score matching

## Abstract

Reading for pleasure has been shown to have benefits for academic attainment and the development of empathy. Yet, whether reading for pleasure is linked with other aspects of children's development remains unclear. *Objective***.** This study examines the association between reading for pleasure and children's psychological and behavioural adjustment at the onset of adolescence. *Method*. We analysed data from 8936 participants in the Millennium Cohort Study, Sweeps 4 (age 7) and 5 (age 11), and used propensity score matching methods to match children who read frequently with children with similar individual, social, familial, and behavioural characteristics who read less often. *Results*. Daily reading for pleasure at age 7 was associated with lower levels of hyperactivity/inattention and better prosocial behaviour at age 11. These results for hyperactivity/inattention were replicated when analysing data specifically from children with a history of hyperactivity/inattention at age 7. Results also show that daily reading for pleasure was associated with lower levels of emotional problems. Results were robust to a range of sensitivity analyses. *Conclusions*. Daily reading for pleasure in childhood is independently associated with better behavioural adjustment at the onset of adolescence. Future studies could explore the potential benefit of interventions to encourage reading.

## Highlights

• Reading for pleasure at age 7 is associated with strengths and difficulties at age 11.
• This association is seen in analyses that control confounding with matched samples.
• Reading is associated with later lower levels of hyperactivity/inattention.
• Reading is related to better prosocial behaviour.
• Reading is associated with lower levels of emotional problems.

## 1 Introduction

This study examines the relationship between reading for pleasure and children's positive and negative psychological and behavioural adjustment. This topic is of importance, given that adjustment problems in childhood tend to persist into adulthood (Case et al., 2005; Currie and Stabile, 2002; Egan et al., 2015; Hofstra et al., 2001) and are linked with social and financial challenges, including youth unemployment (Egan et al., 2015), crime (OECD, 1997), and mental health problems (Williams, 2013).

Over the past decade, there has been a growth of research showing the benefits of reading for prosocial behaviour (Bal and Veltkamp, 2013; Djikic et al., 2013; Johnson, 2012; Mar et al., 2009; Mar and Oatley, 2008; Scales et al., 2000). This research has demonstrated a direct relationship between reading fiction and both empathy and helping behaviours (Clark and Rumbold, 2006; Djikic et al., 2012; Johnson, 2012). One theory for the benefits of reading is that reading simulates emotional states and the corresponding desire to modulate behaviours to suit those states, which supports psychological and behavioural change in real life (Oatley, 1999), and helps readers to understand the emotions of others better (Bal and Veltkamp, 2013; Mar and Oatley, 2008; Oatley, 2012). Reading also stimulates the part of the mind that plans actions in order to achieve goals, supporting behaviour change (Mak and Fancourt, 2020; Oatley, 2012). It has also been suggested that reading may help establish identity and may thus encourage behaviours that then support this identity (Pelowski and Akiba, 2011). Supporting this theory, reading is associated with greater self-confidence as well as more empathetic understanding of other cultures and higher community participation (Clark and Rumbold, 2006).

There is, however, a notable paucity of studies investigating the relationship between reading for pleasure and other aspects of psychological and behavioural adjustment. Therefore, this study explores the longitudinal association between reading and children's adjustment. A central problem when considering this relationship is that reading for pleasure is socially patterned; children who read more frequently may possess characteristics (e.g., higher parental educational levels, higher motivation, and higher levels of human capital) that favour more positive outcomes. While studies experimentally manipulating the frequency of reading among children can be challenging both practically and ethically, this study used propensity score matching (PSM), which mimics an experimental approach using observational data by helping effectively control for selection on observables that might possibly influence the probability of children's reading for pleasure and their psychological and behavioural adjustment.

## 2 Method

### 2.1 Participants

We used data from the Millennium Cohort Study (MCS), a UK nationally-representative, longitudinal study that follows around 19,000 young people who were aged nine months in 2000–2001, with follow-up every few years. This study used data from Sweep 4 (2008) and followed children up at Sweep 5 (2011) when participants were aged 7 and 11 respectively; response rates across Sweeps 4 to 5 were 82% and 81% (Gallop et al., 2013; Gray et al., 2010). A total of 13,857 children were included in Sweep 4 of MCS, in which 13,469 children were followed up at Wave 5. We restricted the sample to exclude those who were born as twins or triplets (to minimise the complexity of the differences between fraternal or dizygotic twins; less than 1.5% of the whole sample was born as twins or triplets) (*n* = 13,287). We also only considered participants with biological parents (*n* = 13,176) because data were unavailable on the mental health of biological parents for those who had been adopted. Of these, 8936 participants provided full data across all measures and thus were included in analyses. A comparison of descriptive statistics between the whole sample and the analytical sample appears in Supplementary Table 1.

MCS has received ethical approval from the NHS Multi-Centre Research Ethics Committee (MREC), and all participants gave informed consent. All methods were performed in accordance with the relevant guidelines and regulations.

### 2.2 Measures

Reading for pleasure was measured at age 7. Parents were asked how often the children read for enjoyment (i.e., not for school). The original indicator was a seven-point scale, ranging from ‘less often or never’, ‘at least once a year’, ‘every few months’, ‘at least once a month’, ‘once or twice a week’, ‘several times a week’ to ‘every day or almost every day’. Due to both the potential inaccuracy of parental-reported reading frequency and to a negative skew on responses (with 39% of parents reporting that their children read every day), we categorised responses into an index of ‘most days’ vs. ‘other.’ To test more extreme responses, we also created an additional binary variable focused on extremes of reading frequency—children who read most days versus those who never read or read less often than once a year (as indicative of rare reading).

Psychological and behavioural adjustment in children was measured using the Strengths and Difficulties Questionnaire (SDQ), a short screening questionnaire of psychosocial problems for children and adolescents based on the Rutter Questionnaires and later revised by Goodman to focus on current child psychopathology (Goodman, 1997). It contains 25 items grouped into five main scales: prosocial behaviour, emotional symptoms, peer relationship problems, conduct problems, and hyperactivity/inattention. It has been suggested that these scales are well-validated (Riso et al., 2010) and are comparable with the Child Behaviour Check List (CBCL), a respected measure used in assessing childhood problems and clinical child psychiatric diagnoses (Goodman et al., 2000; Goodman and Scott, 1999; Stone et al., 2010). The SDQ has also been used in clinical practice (Mullick and Goodman, 2001). We used maternal ratings of their children across the five domains, with the scores measured at ages seven and 11 and standardised to have a mean 0 and a standard deviation of 1. Except for the prosocial behaviour indicator, higher scores indicate a greater incidence of problems.

We used directed acyclic graphs to identify factors that could be associated with both reading for pleasure and children's strengths and difficulties, or with children's strengths and difficulties only based on previous empirical research (Brookhart et al., 2006; Caliendo and Kopeinig, 2008; Rubin, 2001; Sauer and VanderWeele, 2013). Factors (all measured at age 7; our baseline) used for matching include children's gender and ethnicity (White vs. mixed vs. Indian vs. Pakistani and Bangladeshi vs. Black or Black British vs. other ethnic group); and parents' marital status (married/remarried/in a civil partnership vs. single, never married and never in a civil partnership vs. legally separated/divorced/widowed/in a surviving civil partnership), parents' educational levels (no recognised qualifications vs. passed General Certificate of Secondary Education – GCSE – exams with grades D-G vs. passed 4–5 GCSE exams with grades A*-C vs. passed 2 or more Advanced (A-levels) exams vs. higher education (e.g., a Higher Education Certificate/BTEC) vs. further education (e.g., Higher Education Diploma/Foundation Degree)), and employment status (semi-routine and routine vs. lower supervisory and lower technician vs. small employers and self-employed vs. intermediate vs. managerial/professional). Additionally, and importantly, we matched on the mental health condition of parents (Kessler Psychological Distress Questionnaire (K6), standardised) (Kessler et al., 2002), the levels of closeness between parents and children (a four-point scale, ranging from ‘not very close’, ‘fairly close’, ‘very close’, ‘extremely close’, standardised), the frequency of a parent reading to a child (to differentiate from children reading themselves or being read to; a six-point scale measured at age 7, ranging from ‘not at all’, ‘less often than once a month’, ‘once or twice a month’, ‘once or twice a week’, ‘several times a week’, and ‘every day or almost every day’), and children's strengths and difficulties indexes at baseline (all standardised).

### 2.3 Statistics

To compare the outcomes of children who read most days with the outcomes of children who read less often (the ‘counterfactual situation’), we used propensity score matching (PSM), a technique that simulates an experimental setting in an observational data set and creates a treatment group and a control group from the sample (Rosenbaum and Rubin, 1983). PSM controls more effectively than regression approaches for the effects of observed confounders, such that while results remain observational, bias attributable to confounding reduces significantly.

We used PSM to provide an estimate of the difference between the average outcome for children who read most days and the average outcome for the same group under the hypothetical scenario that they read less often (the average treatment effect on the treated; ATT). The ATT for individuals is defined as:

$$\text{ATT}=\text{E}\left({\text{Y}}_{1}|D=1\right)-E\left(Y_{0}|D=1\right)$$

where D is the treatment indicator (reading for pleasure most days), Y_1_ is the outcome if treated (children who read most days), and Y_0_ is the outcome if not treated (children who read less often).

**Fig. 1:**
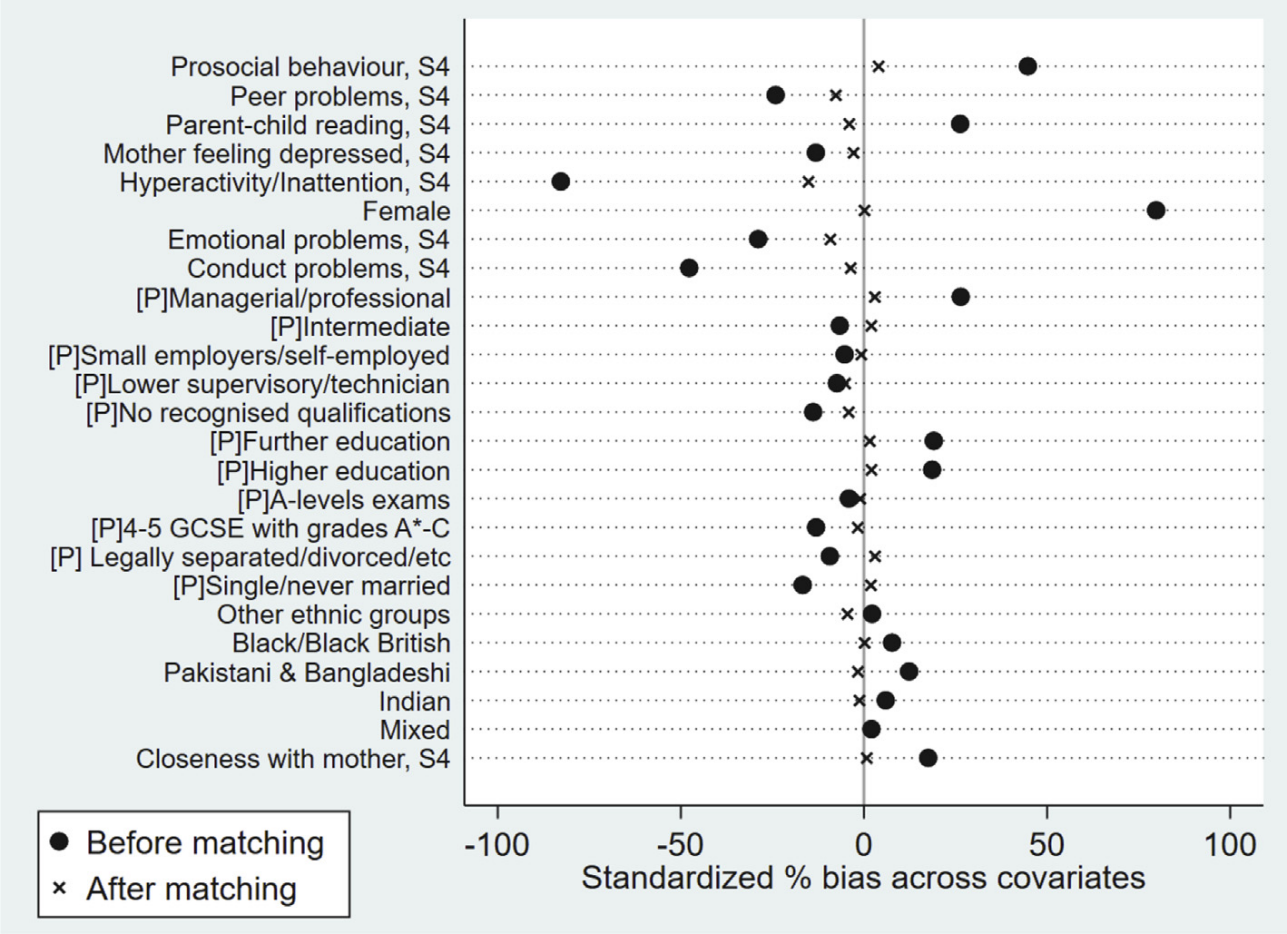
Standardised % bias across covariates, kernel matching.

Specifically, we used unweighted PSM models (Solon et al., 2015; Winship, Christopher; Radbill, 1994) and applied Epanechnikov kernel matching with 0.05 bandwidths, which takes more information from the matches whose propensity scores are closer to each other and down-weights those whose propensity scores are distal from each other (Guo and Fraser, 2015). We imposed the common support condition to ensure the quality of the matches (Caliendo and Kopeinig, 2008). This method led to four observations from the treatment group being dropped due to poor quality of matching. Ninety-five percent confidence intervals were computed using bootstrapping techniques with 100 replications. All estimations handled missing values (i.e., respondents who did not provide a full data across the measures) with list-wise deletion. We achieved a high quality of matching, with unobservable heterogeneity minimised. Nearly all analyses show Rubin's B<25%, Rubin's R of 0.5–2, and a percentage bias of <10% for each covariate (Fig. 1) (Morgan, 2018; Rubin, 2001).

We ran several sets of sensitivity analyses. First, we tested the consistency of the findings when using more extreme frequencies (reading for pleasure most days vs. never/less often than once a year). Second, we additionally matched children not just on aspects of maternal mental health and children's closeness with their mothers but also on their fathers' mental health and the level of closeness between father and child. This matching reduced the sample to 7016 due to missing data from some fathers. Third, it has been shown reading ability affects how much children read outside school (Van Bergen et al., 2018). Thus, to confirm that results were not just driven by ability in reading, we repeated our analysis excluding children with any reading difficulties (rated by the children's mother). Finally, in order to test whether associations were found specifically amongst children with a prior history of poorer behavioural and/or psychological adjustment, we repeated the analyses on the sample who scored in the poorest 40% for each subscale of SDQ.

## 3 Results

### 3.1 Demographics

Of the children in the sample, 49.7% were female, 89.6% of them were white, and 42.9% had parents with higher education qualifications (see Supplementary Table 1). Amongst this sample, there was a significant difference across all variables amongst those children who read most days compared to those who read less often (see Fig. 1 and Supplementary Table 2). Yet, after applying PSM and matching participants who read most days with children who read less often but were equivalent on all identified confounders, these differences were well balanced, such that the only observed difference was in the frequency of reading (Fig. 1).

After matching, groups were well-balanced on all variables, with no between-group differences in demographic backgrounds, relationships with the parents, parental psychological condition, children's previous frequency of reading with parents, and their previous adjustment (Supplementary Table 2).

### 3.2 Hyperactivity/inattention

Amongst the matched sample, the clearest finding was that reading for pleasure most days at age 7 was significantly related to lower hyperactivity/inattention at age 11 (ATT = −0.05, 95% *CI* = −0.09, −0.02, *p* < 0.001) (Table 1). This result was found when matching on all identified confounders including gender, ethnicity, parental marital status, parental educational levels, parental employment status, mother's mental health, levels of mother-child closeness, mother-child frequency of reading together, and children's baseline SDQ. When testing more distinct reading groups and comparing children who read most days with those who never read or read less often than once a year, the magnitude of this association became greater (ATT = −0.20, 95% *CI* = -0.29, −0.12, *p* < 0.001). The finding was also consistent when matching additionally on fathers' data (Table 1), when excluding children with any reading difficulty (Table 2), and when restricting the sample to children in the top 40% for hyperactivity problems (Table 3).

**Table 1:** Reading for pleasure (age 7) and SDQ (age 11).

Frequency of reading for pleasure	Strengths and difficulties	Matched on mother's data only		Matched on both mother's and father's data	
		ATT (95% *CI*)	*p*	ATT (95% *CI*)	*p*
*Most days vs. any other frequency*	Hyperactivity/Inattention	−0.05 (−0.09, −0.02)	<0.001	−0.06 (−0.10, −0.02)	0.005
	Prosocial behaviour	0.04 (0.01, 0.08)	0.020	0.05 (0.01, 0.09)	0.023
	Emotional problems	0.01 (−0.03, 0.04)	0.721	0.00 (−0.04, 0.05)	0.952
	Peer problems	0.03 (−0.00, 0.07)	0.059	0.04 (−0.00, 0.08)	0.078
	Conduct problems	−0.00 (−0.04, 0.03)	0.824	−0.01 (−0.05, 0.03)	0.566
	Mean bias	0.7		0.8	
	Rubin's *B*	4.0		4.8	
	Rubin's *R*	1.06		1.06	
	*N*	8936		7016	
*Most days vs. never/less often than once a year*	Hyperactivity/Inattention	−0.20 (−0.29, −0.12)	<0.001	−0.21 (−0.32, −0.11)	<0.001
	Prosocial behaviour	0.09 (0.01, 0.17)	0.035	0.12 (0.03, 0.20)	0.010
	Emotional problems	−0.11 (−0.21, −0.02)	0.021	−0.13 (−0.25, −0.00)	0.043
	Peer problems	−0.06 (−0.18, 0.05)	0.283	−0.08 (−0.20, 0.04)	0.215
	Conduct problems	−0.02 (−0.10, 0.05)	0.541	−0.04 (−0.13, 0.05)	0.349
	Mean bias	3.8		4.0	
	Rubin's *B*	25.7		25.4	
	Rubin's *R*	1.12		1.30	
	*N*	4479		3564	

*Note*. Columns present ATT estimates from PSM models using Epanechnikov kernel matching with 0.05 bandwidths; common support condition is imposed. The models controlled all covariates. ATT Ninety-five percent confidence intervals in parentheses were computed by bootstrapping with 100 replications. Success of the propensity score matching was assessed using a percentage bias of <10% for each covariate, Rubin's *B* <25% and Rubin's *R* of 0.5–2. Models controlled for children's gender and ethnicity; parents' marital status, educational levels, and employment status; mental health condition of parents; the levels of closeness between parents and children; parent-child reading engagement frequency; and children's strengths and difficulties indexes at baseline.

**Table 2:** Reading for pleasure (age 7) and SDQ (age 11) among children with no reading difficulty.

	ATT (95% *CI*)	*p*
Strengths and difficulties		
Hyperactivity/Inattention	−0.04 (−0.07, −0.00)	0.028
Prosocial behaviour	0.05 (0.01, 0.09)	0.018
Emotional problems	0.02 (−0.03, 0.07)	0.387
Peer problems	0.04 (−0.00, 0.08)	0.056
Conduct problems	−0.00 (−0.04, 0.04)	0.952
Mean bias	0.6	
Rubin's *B*	4.2	
Rubin's *R*	1.08	
*N*	6855	

*Note*. Columns present ATT estimates from PSM models using Epanechnikov kernel matching with 0.05 bandwidths; common support condition is imposed. The models controlled all covariates. ATT 95% confidence intervals in parentheses were computed by bootstrapping with 100 replications. Success of the propensity score matching was assessed using a percentage bias of <10% for each covariate, Rubin's *B* <25%, and Rubin's *R* of 0.5–2. Models controlled for children's gender and ethnicity; parents' marital status, educational levels, and employment status; mental health condition of parents; the levels of closeness between parents and children; parent-child reading engagement frequency; and children's strengths and difficulties indexes at baseline.

**Table 3:** Reading for pleasure (age 7) and SDQ (age 11) among the top 40% psychological and behavioural issues.

	Most days vs. Any other frequency	
	ATT (95% *CI*)	*p*
Strengths and difficulties		
Hyperactivity/Inattention	−0.12 (−0.20, −0.04)	0.005
Mean bias	0.7	
Rubin's B	3.9	
Rubin's R	1.13	
*N*	3539	
Prosocial behaviour	0.08 (-0.01, 0.16)	0.077
Mean bias	0.9	
Rubin's B	5.4	
Rubin's R	1.20	
*N*	2792	
Emotional problems	−0.07 (−0.13, 0.00)	0.046
Mean bias	0.7	
Rubin's B	3.9	
Rubin's R	1.11	
*N*	3858	
Peer problems	−0.01 (−0.08, 0.07)	0.871
Mean bias	0.8	
Rubin's B	3.6	
Rubin's R	1.08	
*N*	3935	
Conduct problems	−0.06 (−0.14, 0.01)	0.100
Mean bias	0.8	
Rubin's B	4.5	
Rubin's R	1.14	
*N*	3311	

*Note*. Columns present ATT estimates from PSM models using Epanechnikov kernel matching with 0.05 bandwidths; common support condition is imposed. The models controlled all covariates. ATT 95% confidence intervals in parentheses were computed by bootstrapping with 100 replications. Success of the propensity score matching was assessed using a percentage bias of <10% for each covariate, Rubin's B<25%, and Rubin's R of 0.5–2. Models controlled for children's gender and ethnicity; parents' marital status, educational levels, and employment status; mental health condition of parents; the levels of closeness between parents and children; and parent-child reading engagement frequency.

### 3.3 Prosocial behaviour

Reading for pleasure was associated with higher levels of prosocial behaviour (ATT = 0.04, 95% *CI* = 0.01, 0.08, *p* = 0.020). When comparing children who read most days with those who never read or read less often than once a year (a more distinct group), the magnitude of this association also became greater. The finding was also consistent when matching additionally on fathers’ data (Table 1) and when excluding children with any reading difficulty (Table 2).

### 3.4 Emotional problems

Reading for pleasure most days of the week at age 7 was associated with fewer emotional problems at age 11, but only when comparing the more distinct groups (ATT = −0.11, 95% *CI* = −0.21, −0.02, *p* = 0.021). Nevertheless, this finding held when taking into account fathers’ mental health (ATT = −0.13, 95% *CI* = −0.25, −0.00, *p* = 0.043) and when restricting the sample to those in the highest 40% for emotional problems (ATT = −0.07, 95% *CI* = −0.13, 0.00, *p* = 0.046). Yet, no association was found between reading for pleasure and later emotional problems when comparing children who read most days with those with other frequency or when excluding children with any reading difficulty.

### 3.5 Peer problems

There was no clear association between reading and peer problems across any analyses. Although reading for pleasure most days of the week at age 7 had a slight association with greater peer problems at age 11 (ATT = 0.03, 95% *CI* = −0.00, 0.07, *p* = 0.059), this finding was attenuated in all further analyses.

### 3.6 Conduct problems

There was no association between reading and conduct problems across any analyses.

## 4 Discussion

This study explored the association between reading for pleasure and children's psychological and behavioural adjustment. Using matched pairs from the MCS, a rich nationally-representative data set, we found a longitudinal relationship between reading at age 7 and lower levels of hyperactivity/inattention at age 11. We also found associations between reading for pleasure and higher levels of prosocial behaviours amongst children as well as some suggestions that reading for pleasure most days could be linked with lower emotional problems. Although the relationship appears modest (reading accounts for less than one fourth of a standard deviation in behaviours), it may still be meaningful at a population level.

Our main finding was a strong and consistent relationship between reading and hyperactivity/inattention across all models. As might be expected, the association was most prominent when the reading groups were more distinct, with strongest findings when comparing reading most days with reading never or rarely. A key question is whether the relationship we saw indicates that reading for pleasure could causally lead to lower hyperactivity/inattention problems or whether it could be a result of reverse causality or confounding factors. Whilst our analyses are of observational data and therefore causality cannot be assumed, the richness of the data set allowed respondents to be matched vertically on a large set of covariates, which minimised the risk of bias caused by unobserved heterogeneity. As a result, respondents from the treatment group shared almost identical backgrounds with those from the control group on all identified confounding factors, suggesting that at least part of the estimated relationship operated in the assumed direction. Further, it is notable that our results were found not just amongst the overall sample but also amongst those in the top 40% of hyperactivity problems at age 7. This finding suggests that the association is not merely an artefact of those children without hyperactivity problems at age 7 being more likely to have the attention span to read. Instead, it is possible that reading for pleasure might promote longer attention span, stimulating concentration and more stable behaviours. Moreover, while transitioning into early adolescence (around the age of 10–14), reading books that are relevant to the new experience of adolescence may offer a source of comfort for children and normalise experiences. Additionally, research on preschool children found that the home reading environment fosters brain development, which could potentially help with hyperactivity/inattention both at this age and at the onset of adolescence (Hutton et al., 2015). Overall, this potential protective association between reading and hyperactivity is important given that hyperactivity/inattention is related to long-term poorer educational attainment, substance use disorders, and poorer quality of life (Danckaerts et al., 2010; Hechtman et al., 2016; Pingault et al., 2011). Future studies could explore whether the behavioural differences noted here are accompanied by underlying differences in brain development or morphology over time and ascertain further whether reading for pleasure could be a valuable novel intervention within ADHD treatment protocols for children.

In line with the previous studies (Bal and Veltkamp, 2013; Djikic et al., 2013), another key and consistent finding from this study was that reading for pleasure was associated with increased prosocial behaviour amongst children. This finding is supported by the theory of mind, which suggests that reading facilitates individuals' ability to understand others’ feelings and emotions (Shamay-Tsoory and Aharon-Peretz, 2007) and thus increases their development in empathy and prosocial behaviour. Indeed, it has been suggested that stories be utilised more in schools for promoting and encouraging socially responsible behaviours in children (Binnendyk and Schonert-Reichl, 2002).

Our analysis also showed that reading was associated with less emotional problems, although this finding was much less consistent across our different analyses. It is possible that, along with enhancing empathy, reading may also stimulate other positive emotions that support the development of a more positive sense of self (Oatley, 2012). It is also plausible that reading books may provide sources of escapism and fantasy, allowing children to distract themselves from outside influences, including negative emotions. Another potential explanation is that reading for pleasure may reduce children's time spent on activities that may cause psychological distress, such as watching TV or interacting with social media (Hamer et al., 2009; Sampasa-Kanyinga and Lewis, 2015). These findings are promising because poor prosocial behaviours and emotional problems in children and young people are known to strongly correlate with substance use/misuse and delinquency, with these associations likely to persist into adulthood and be linked with a higher probability of developing mental health problems (NICE, 2013). Nevertheless, this result remains to be explored further to determine its reproducibility.

### 4.1 Strengths and limitations

Our study has several strengths. This study is the first to examine the association between reading for pleasure and children's strengths and difficulties. It was based on a nationally representative sample from a very rich data set, which also provided an ideal opportunity for us to control for a large set of confounding factors when using the PSM approach. Yet, the study also had several limitations. First, our study was observational, and unobserved confounding factors could still explain the associations found. Thus, it remains for future intervention studies to confirm whether results are causal. Second, whilst we know the frequency with which children read for pleasure, it is unclear what their motivation for reading was. Future studies could explore whether different types of motivation lead to differential responses. Further, although we tested different thresholds for dividing the sample into differing frequencies of reading, future research could help to ascertain more clearly what frequency of reading (whether daily or just a few times a week) is associated with behavioural adjustment. Future studies should also investigate how trajectories of reading across childhood are associated with later development in adolescence and adulthood. Finally, while we focused on reading for pleasure, the type of writing (e.g., fiction vs. non-fiction) was not considered in this study due to unavailable data from the data set. It has been suggested that fiction literature may have a greater influence on readers' emotions and behaviour than non-fiction literature (Djikic et al., 2013). Further studies that consider the nature of books will need to be undertaken.

## 5 Conclusions

Our findings suggest that reading for pleasure most days in childhood is associated with better behavioural adjustment at the onset of adolescence. Given known challenges in promoting positive psychological and behavioural development in childhood and in particular across sensitive transition periods such as the onset of adolescence, these results suggest the value in considering measures of reading for pleasure for studies that are interested in children's and adolescents' emotional and behavioural development, as well as undertaking longitudinal experimental studies to explore the impact of encouraging reading amongst children on their future psychological and behavioural adjustment.

## CRediT authorship contribution statement

**Hei Wan Mak:** Methodology, Software, Formal analysis, Investigation, Writing - original draft. **Daisy Fancourt:** Conceptualization, Supervision, Writing - review & editing.
