# Supplementary material for: Longitudinal associations between reading for pleasure and child maladjustment: Results from a propensity score matching analysis
Source: Soc Sci Med. 2020 May;253:112971. doi: 10.1016/j.socscimed.2020.112971 (PMC7429985; doi:10.1016/j.socscimed.2020.112971)
Supplement: Multimedia component 1 [file mmc1.docx]

| **Supplementary Table 1**  *Descriptive statistics of the whole and analytical samples.* | | | |
| --- | --- | --- | --- |
|  | | **Mean (*SD*) or %** | |
|  | | **Complete sample (raw data)** | **Analytical sample (*N =* 8,936)** |
| **Strengths and difficulties^1^, Sweep 4** | Hyperactivity/inattention (ranges from 5-15) | 8.33 (2.51)  *N =* 12,755 | 8.09 (2.42) |
|  | Prosocial behaviour (ranges from 5-15) | 13.6 (1.61)  *N =* 13,108 | 13.7 (1.54) |
|  | Emotional problems (ranges from 5-15) | 6.50 (1.74)  *N =* 12,815 | 6.37 (1.62) |
|  | Peer problems (ranges from 5-15) | 6.18 (1.49)  *N =* 11,921 | 6.01 (1.39) |
|  | Conduct problems (ranges from 5-15) | 6.35 (1.51)  *N =* 12,965 | 6.18 (1.38) |
| **Strengths and difficulties, Sweep 5** | Hyperactivity/inattention (ranges from 5-15) | 8.08 (2.46)  *N =* 11,866 | 7.87 (2.37) |
|  | Prosocial behaviour (ranges from 5-15) | 13.8 (1.54)  *N =* 12,030 | 13.9 (1.45) |
|  | Emotional problems (ranges from 5-15) | 6.85 (1.98)  *N =* 11,938 | 6.72 (1.91) |
|  | Peer problems (ranges from 5-15) | 6.34 (1.66)  *N =* 11,633 | 6.19 (1.40) |
|  | Conduct problems(ranges from 5-15) | 6.35 (1.53) *N =* 11,936 | 6.19 (1.40) |
| **Demographic variables** | Female | 38.6  *N =* 19,244 | 49.7 |
|  | White | 83.7  *N =* 11,598 | 89.6 |
|  | Mixed | 2.73  *N =* 378 | 2.32 |
|  | Indian | 2.48  *N =* 343 | 2.10 |
|  | Pakistani and Bangladeshi | 6.41  *N =* 888 | 3.21 |
|  | Black or Black British | 3.31  *N =* 459 | 1.97 |
|  | Other ethnic group (incl. Chinese, other) | 1.38  *N =* 191 | 0.85 |
|  | [P] Married/remarried/in a civil partnership | 65.8  *N =* 9,076 | 73.9 |
|  | [P] Single, never married and never in a civil partnership | 22.1  *N =* 3,042 | 16.4 |
|  | [P] Legally separated/divorced/widowed/in a surviving civil partnership | 12.1 *N =* 1,674 | 9.71 |
| **Education** | [P] No recognised qualifications | 16.5  *N =* 2,287 | 9.49 |
|  | [P] Basic qualifications at age 16 (passed 3-4 General Certificate of Secondary Education – GCSE- exams with grades D-G) | 6.86  *N =* 950 | 5.55 |
|  | [P] Further qualifications at age 16 (passed 4-5 GCSE exams with grades A*-C) | 26.2  *N =* 3,631 | 26.0 |
|  | [P] Qualifications at age 18 (passed 2 or more Advanced (A-level) exams) | 15.0  *N =* 2,077 | 16.0 |
|  | [P] Higher education (e.g., a Higher Education Certificate/BTEC) | 26.7  *N =* 3,697 | 32.0 |
|  | [P] Further higher education (e.g., Higher Education Diploma or Foundation Degree) | 8.76  *N =* 1,214 | 10.9 |
| **Social status** | [P] Semi-routine and routine | 23.5  *N =* 2,580 | 21.5 |
|  | [P] Lower supervisory and lower technician | 10.9  *N =* 1,203 | 10.9 |
|  | [P] Small employers and self-employed | 15.3  *N =* 1,685 | 15.0 |
|  | [P] Intermediate | 8.90  *N =* 978 | 8.89 |
|  | [P] Managerial/ professional | 41.4  *N =* 4,546 | 43.68 |
| **Relationships with parents, Sweep 4** | Closeness with mother^2^ | 1. (1.00)   *N =* 13,004 | 0.02 (0.98) |
| **Parents’ depression in the past 30 days, Sweep 4** | Mother feeling depressed^2^ | -0.00 (1.00)  *N =* 13,073 | -0.11 (0.87) |
| **Previous reading behaviour with parents, Sweep 4** | Parent-child reading engagement^3^ | 5.07 (1.11)  *N =* 13,803 | 5.21 (0.98) |
| *Note*. The table shows means and standard deviations in parentheses or percentages. [P] represents parent. ^1^Each SDQ domain has 5 items; each item consists of a 3-point scale: not true; somewhat true; and certainly true. ^2^Higher scales indicate a greater level of closeness/feeling depressed. ^3^A 6-point scale: not at all; less often than once a month; once or twice a month; once or twice a week; several times a week; and every day or almost every day. | | | |

| **Supplementary Table 2**  **Descriptive statistics: average value of outcome and control variables before and after matching** | | | | | | | | | |  |
| --- | --- | --- | --- | --- | --- | --- | --- | --- | --- | --- |
|  | **Before matching** | | | **After matching** | | | | | | |
|  | **Children who read most days** | **Children who read less often than most days** | ***p*** | | **Children who read most days** | **Children who read less often than most days** | | ***p*** | |  |
| ***Strengths and difficulties, Sweep 4*** | |  |  |  | | |  | |  | |
| Hyperactivity/inattention | -0.308 | 0.052 | *** | -0.303 | | | -0.286 | |  | |
| Prosocial behaviour | 0.182 | -0.019 | *** | 0.183 | | | 0.168 | |  | |
| Emotional problems | -0.117 | -0.065 | ** | -0.118 | | | -0.114 | |  | |
| Peer problems | -0.122 | -0.071 | * | -0.123 | | | -0.119 | |  | |
| Conduct problems | -0.233 | -0.032 | *** | -0.233 | | | -0.229 | |  | |
| ***Demographic variables*** |  |  |  |  | | |  | |  | |
| Female | 0.622 | 0.410 | *** | 0.621 | | | 0.620 | |  | |
| Mixed^1^ | 0.024 | 0.022 |  | 0.024 | | | 0.024 | |  | |
| Indian | 0.020 | 0.022 |  | 0.020 | | | 0.020 | |  | |
| Pakistani and Bangladeshi | 0.032 | 0.032 |  | 0.031 | | | 0.031 | |  | |
| Black or Black British | 0.019 | 0.020 |  | 0.019 | | | 0.021 | |  | |
| Other ethnic group (incl. Chinese, other) | 0.008 | 0.009 |  | 0.008 | | | 0.009 | |  | |
| [P] Single, never married and never in a civil partnership^2^ | 0.152 | 0.172 | * | 0.151 | | | 0.154 | |  | |
| [P] Legally separated/divorced/widowed/in a surviving civil partnership | 0.092 | 0.101 |  | 0.092 | | | 0.089 | |  | |
| ***Socio-economic status*** |  |  |  |  | | |  | |  | |
| [P] No recognised qualifications^3^ | 0.078 | 0.107 | *** | 0.078 | | | 0.077 | |  | |
| [P] Further qualifications at age 16 (passed 4-5 GCSE grades A*-C) | 0.230 | 0.282 | *** | 0.230 | | | 0.227 | |  | |
| [P] Qualifications at age 18 (passed 2 or more Advanced (A-level) exams) | 0.158 | 0.162 |  | 0.158 | | | 0.156 | |  | |
| [P] Higher education (e.g., a Higher Education Certificate/BTEC) | 0.354 | 0.297 | *** | 0.354 | | | 0.360 | |  | |
| [P] Further higher education (e.g., Higher Education Diploma/ Foundation Degree) | 0.130 | 0.093 | *** | 0.130 | | | 0.130 | |  | |
| [P] Lower supervisory and lower technician^4^ | 0.099 | 0.117 | ** | 0.099 | | | 0.100 | |  | |
| [P] Small employers and self-employed | 0.141 | 0.157 | * | 0.141 | | | 0.140 | |  | |
| [P] Intermediate | 0.084 | 0.092 |  | 0.084 | | | 0.085 | |  | |
| [P] Managerial/ professional | 0.477 | 0.408 | *** | 0.477 | | | 0.480 | |  | |
| ***Relationships with parent, Sweep 4*** | | |  |  | | |  | |  | |
| Closeness with mother^5^ | 0.067 | -0.013 | *** | 0.067 | | | 0.067 | |  | |
| ***Parent’s depression in the past 30 days, Sweep 4*** | | |  |  | | |  | |  | |
| Mother feeling depressed^5^ | -0.138 | -0.094 | * | -0.141 | | | -0.125 | |  | |
| ***Previous reading behaviour with parent, Sweep 4*** | | | |  | | |  | |  | |
| Reading for enjoyment, not for school^6^ | 5.356 | 5.101 | *** | 5.356 | | | 5.344 | |  | |
| *Note*. The table shows means and standard deviations in parentheses or percentages.  Statistical significance is denoted by asterisks: * sig at 5%, ** sig at 1%, *** sig at 0.1%.  [P] represents parent. Reference groups: ^1^White; ^2^[P] Married/remarried/in a civil partnership; ^3^[P] Basic qualifications at age 16 (passed 3-4 General Certificate of Secondary Education – GCSE- exams with grades D-G); ^4^[P] Semi-routine and routine. ^5^Higher scales indicate a greater level of closeness/feeling depressed. ^6^A 6-point scale: not at all; less often than once a month; once or twice a month; once or twice a week; several times a week; and every day or almost every day. | | | | | | | | | |  |
